# Coughs

**Published:** 1886-02-20

**Authors:** R. C. Word

**Affiliations:** Professor of Physiology in the Southern Medical College, Atlanta, Ga.


### Southern Medical Record.

A MONTHLY JOURNAL OF PRACTICAL MEDICINE.

☞All Communications must be addressed to R. C. WORD, M. D., Managing Editor.

"*Quicquid Præcipies Esto Brevis.*"

VOL. XVI. ATLANTA, GA., FEBRUARY 20, 1886. No. 2.

### ORIGINAL AND SELECTED ARTICLES.

### COUGHS.

Remarks made at a Clinic by R. C. WORD, M. D., Professor of Physiology in the Southern Medical College, Atlanta, Ga.

A cough is a more or less violent expiration succeeding to a deep inspiration, the air being forced out by the action of the thoracic and abdominal expiratory muscles, by which any irritating matter is expelled through the bronchi and largynx into the mouth.

A cough is not, in every case, caused by the presence of irritating or foreign matter in the air passages ; it not infrequently results from reflex influences, seated in remote parts, in which case there is no sputa or expectoration, and the cough is dry. "The nine-months cough," as it is called—often observed in pregnant women—is of this class. In disorders of the liver and stomach a dry, hacking cough is not uncommon. Irritation of the internal ear produces a cough of this kind.

A reflex cough, and indeed any kind of cough, is not itself a disease, but a symptom merely of irritation, either direct or reflex, in the lungs or air passages.

A cough may constitute a pathognomonis symptom of disease, as in croup. Here the cough is of a stridulous barking character, and is always recognized as the croup cough ; so of the characteristic whoop of the whooping cough. In pleuresy, we have a short, restrained cough, causing acute pain. In insipient tuberculosis, there is a dry, hacking cough, caused by the irritation of tuberculous matter in the lung tissue. In advanced phthisis, where the tubercles are breaking down, the cough becomes loose and rattling from the presence of pus and mucous in bronchi and air cells. At early morn the phthisical cough is more violent, and the expectoration freer and more copious as a result of the accumulation during the hours of sleep.

The cough of acute bronchitis is the result of catarrhal irritation or inflammation in the mucous membrane lining the bronchi. Usually, it is confined to the larger bronchi. The symptoms are those of a common cold, ushered in by sneezing, chilly sensations, more or less hoarseness, and cough. The throat is often dry and sore, and a flush or fever and headache is commonly present ; pain-tightness is felt beneath the sternum in the upper part of the chest, with sense of oppression and, perhaps, dyspnœa. During the early part of the acute stage the cough is apt to be deep, harsh, and violent, with little or no expectoration ; a small particle of tough sputa, sometimes producing a prolonged and painful effort to dislodge it. The straining thus attending the cough sometimes causees the rupture of small vessels in the throat or air passages, causing the sputa to be stained with blood ; after a time, however, the more acute symptoms subside, and the secretion in the bronchi becomes more free and less tenacious, and the cough is not so straining to the patient.

When the inflammation is lower down in the smaller bronchial tubes, it is called capillary bronchitis, and is a more intractable form of the disease. Here the cough is deep seated, and almost constant, and the dyspnœa and oppression much greater, and the sputa is viscid and ropy, or may become purulent.

But it is not my object to describe minutely the various kinds of cough, but, rather, to submit a few formulæ of cough remedies which will be useful in practice.

It is a generally accepted fact that when a cough is tight and the expectoration scant, the case is not doing well ; and we should use remedies calculated to relax the organs and encourage the secretion from the congested and inflamed mucous follicles in the epithelial lining of the air passages. In milder cases, it may be sufficient to give a saline cathartic, followed by warm drinks, and diaphoretics, seeking thus, by diversion to the bowels and skin, to equalize the circulation and relieve the hyperæmia or undue circulation of the blood in the broncial mucous membrane. This result may be often promptly accomplished without a purgation, by keeping the patient warm in bed and administering a full dose of quinine and Dover's powder If there be considerable fever, and the opiate fail to produce satisfactory diaphoresis, 1-drop doses of aconite, every two or three hours, will be found to reduce the fever and bring on the sweating, with great relief to all the symptoms.

In lieu of this, the following cough mixture may be used with advantage :

℞. Com. syrup of squills.........................℥ j,

Syrup of ipecac.............................℥ j,

Syrup of tar..................................℥ ij. M.

Sig. One teaspoonful every two hours ; or the following :

℞. Tinct. aconite..............................gtt. xv,

Camph. tinct. of opii........................ʒ iv,

Syrup of ginger.........................℥ iss,

Spirits of nitre, to make....................℥ ij. M.

The syrup of tar, having, in a milder and more pleasant form, all the properties of turpentine, is a good remedy in itself, and makes a fine vehicle with which to combine other drugs ; as squills, tolu, lobelia, etc. As a lubricating agent, to an inflamed throat, it is excellent, and its benefits to colds are enhanced by its gentle diuretic action. Combined with lobelia, it is a good remedy in coughs, especially with asthmatic complication ; as—

℞. Syrup of tar........ }

Syrup of tolu....... }

Syrup of lobelia..... } aa.....................℥ j. M.

Syrup of ipecac..... }

Sig. Dessertspoonful every two to four hours.

Or—

℞. Syrup of tar............. }

Fluid ext. wild cherry.... } aa..............℥ I,

Syrup of lobelia .........................℥ j. M.

Sig. Dessertspoonful every three hours.

Opiates are not added to the above, because their continuous use locks the bowels and tends to counteract the influence of the other remedies in loosening the secretions. If, however, there be much pain, or if there be restlessness at night, a portion of morphia or paregoric may be added occasionally, as required.

Sometimes the anodyne influence of the bromides may be better used for this purpose, particularly in children.

In some cases where opium is objectionable, chloral may also be used to promote rest. Of the bromides, the ammonium salt is preferred, as it is known that ammonia is a useful drug in coughs. Three to five grains of the carbonate may be used with fine results in coughs when the patient is feeble ; as in the latter stages of pneumonia, or in other low conditions. It tends to support the strength of the patient, while it also softens the secretions and encourages the expectoration.

℞. Carbonate of ammonia........................grs. xx,

Syrup of ipecac..................................ʒ ij,

Paregoric.........................................ʒ j,

Syrup of wild cherry, to make..............℥ ij. M.

Sig. One teaspoonful every two to three hours.

Antimonial wine, in small and oft repeated doses, is a good remedy to liquify the secreted matters and make easy the expectoration. Iodide of potassium, in 4-grain doses, every three to four hours, is useful for the same purpose.

In chronic phthisical coughs the following is a good formula:

℞. Holland gin (Schnapps)....................℥ vi,
Tinct. nux vomica..............................gtt. lx,
Syrup of tolu.....................................℥ ij,
Creasote.............................................gtt. xx. M.

Sig. From half to 1 tablespoonful, three to four times a day.

Or the following:

℞. Water................................................℥ xij,

Chlorate of potas...............................ʒ j,

Creasote.............................................gtt. xxiv,

Tinct. nux vomica...............................ʒ j. M.

Sig. Tablespoonful three times a day. This is especially useful where the digestion is imperfect.

